# Phase I trial and tumour localisation of the anti-EGFR monoclonal antibody ICR62 in head and neck or lung cancer.

**DOI:** 10.1038/bjc.1996.40

**Published:** 1996-01

**Authors:** H. Modjtahedi, T. Hickish, M. Nicolson, J. Moore, J. Styles, S. Eccles, E. Jackson, J. Salter, J. Sloane, L. Spencer, K. Priest, I. Smith, C. Dean, M. Gore

**Affiliations:** Section of Immunology, McElwain Laboratories, Institute of Cancer Research, Sutton, UK.

## Abstract

**Images:**


British Journal of Cancer (1996) 73, 228-235
© 1996 Stockton Press All rights reserved 0007-0920/96 $12.00

### Phase I trial and tumour localisation of the anti-EGFR monoclonal antibody ICR62 in head and neck or lung cancer

H Modjtahedi[1], T Hickish[2], M Nicolson[2], J Moore[2], J Styles[1], S Eccles[1], E Jackson[1], J Salter[3], J Sloane[3], L Spencer[2], K Priest[2], I Smith[2], C Dean[1] and M Gore[2]

*[1]Section of Immunology, McElwain Laboratories, Institute of Cancer Research, Sutton SM2 5NG, UK; [2]Section of Medicine, and [3]Department of Histopathology, Royal Marsden Hospital, London SW3 6JJ and Sutton, Surrey SM2 5PT, UK.*

**Summary** The purpose of this study was to determine the effect of the first rat monoclonal antibody (MAb ICR62) to the epidermal growth factor receptor (EGFR) in a phase I clinical trial in patients with unresectable squamous cell carcinomas. This antibody effectively blocks the binding of EGF, transforming growth factor (TGF)-α and HB-EGF to the EGFR, inhibits the growth *in vitro* of tumour cell lines which overexpress the EGFR and eradicates such tumours when grown as xenografts in athymic mice. Eleven patients with squamous cell carcinoma of the head and neck and nine patients with squamous cell carcinoma of the lung, whose tumours expressed EGFR, were recruited. Groups of three patients were treated with 2.5 mg, 10 mg, 20 mg or 40 mg of ICR62 and a further eight patients received 100 mg. All patients were evaluated for toxicity using WHO criteria. Patients' sera were tested for the clearance of MAb ICR62 and the development of human anti-rat antibodies (HARA). No serious (WHO Grade III-IV) toxicity was observed in patients treated with up to 100 mg of antibody ICR62. Antibody ICR62 could be detected at 4 h and 24 h in the sera of patients treated with 40 mg or 100 mg of ICR62. Only 4/20 patients showed HARA responses (one at 20 mg, one at 40 mg and two at 100 mg doses) and of these only the former two were anti-idiotypic responses. In four patients receiving doses of ICR62 at 40 mg or greater, biopsies were obtained from metastatic lesions 24 h later and examined for the localisation of ICR62 using anti-rat antibody reagent. In these patients we showed the localisation of MAb ICR62 to the membranes of tumour cells; this appeared to be more prominent at the higher dose of 100 mg. On the basis of these data we conclude that MAb ICR62 can be administered safely to patients with squamous cell carcinomas and that it can localise efficiently to metastases even at relatively low doses.

**Keywords:** monoclonal antibody; epidermal growth factor receptor; squamous cell carcinoma; phase I trial; tumour localisation

It is becoming increasingly evident that alterations in the level of expression of growth factors and their receptors may play an important role in the pathogenesis of human malignancies (Goustin *et al.*, 1986; Aaronson, 1991; Cross and Dexter, 1991; Waterfield, 1991; Pusztai *et al.*, 1993; Mendelsohn and Lippman, 1993; Nicolson, 1993). Of the systems studied, it is clear that the aberrant expression of the epidermal growth factor (EGF) receptor system is particularly important in the development of certain types of human malignancy (Thompson and Gill, 1985; Ozanne *et al.*, 1986; Gullick, 1991; Harris, 1994; Modjtahedi and Dean, 1994). The human EGF receptor is a 170 kDa transmembrane tyrosine kinase that transmits the mitogenic actions of the EGF family of ligands. This includes EGF, transforming growth factor (TGF)-α, amphiregulin, HB-EGF, and betacellulin (Cohen, 1962; Todaro *et al.*, 1976; Das *et al.*, 1977; Shoyab *et al.*, 1989; Higashiyama *et al.*, 1991; Shing *et al.*, 1993; Carpenter, 1987).

Overexpression of the EGFR accompanied by production of one or more of its ligands has been reported in a wide range of human malignancies, including cancer of the bladder, brain, head and neck, pancreas, lung, breast and ovary (Cowley *et al.*, 1984; Ozanne *et al.*, 1986; Neal *et al.*, 1990; Gullick, 1991; Modjtahedi and Dean, 1994). This in turn has been associated with poor survival in many of these patients (for review see Modjtahedi and Dean, 1994). Recent studies have also indicated that the growth of tumours overexpressing the EGF receptor can be stimulated by its ligands in several ways, e.g. via autocrine, paracrine or juxtacrine modes (Sporn and Roberts, 1985; Derynck, 1992; Modjtahedi and Dean, 1994). However, since the ligand-induced activation of these cells occurs primarily via receptors on the cell surface rather than intracellularly, this system may form a suitable target for monoclonal antibody-directed therapy (Van de Vijvert *et al.*, 1991; Baselga and Mendelsohn, 1994; Modjtahedi and Dean, 1994).

We have reported recently the production and characterisation of a number of rat MAbs directed against the extracellular domain of the EGFR using as immunogens the human head and neck tumour cell line HN5, breast carcinoma cell line MDA-MB 468 or the vulval carcinoma cell line A431, all of which overexpress the human EGFR (see Modjtahedi and Dean, 1994 for details). Our aim was to obtain a diverse population of antibodies that bound to distinct epitopes on the EGFR and which were of different isotype in order to select the best MAb or combination of MAbs for therapeutic and diagnostic use. Of these, the IgG2b MAb ICR62 was prominent in that it (a) effectively blocks the binding of EGF, TGF-α, and HB-EGF to EGFR; (b) inhibits the growth of EGFR-overexpressing tumour cell lines *in vitro* (Modjtahedi *et al.*, 1993*a*; Modjtahedi and Dean 1995); and (c) was the most effective antibody in our MAb library for eradicating such tumours in athymic mice (Modjtahedi *et al.*, 1993*b*; Dean *et al.*, 1994). We have also reported recently that antibodies to the EGFR that inhibit the growth of EGFR-overexpressing tumours do so by inducing terminal differentiation and that a further therapeutic effect may be obtained via immunological mechanisms with rat IgG2b MAbs such as ICR62 (Modjtahedi *et al.*, 1994).

For these reasons we have selected MAb ICR62 for a phase I clinical study in patients with unresectable squamous cell carcinoma of the head and neck or lung. These tumour types commonly overexpress the EGFR. With only limited quantities of antibody available, the aims of this initial study

Correspondence: C Dean, Institute of Cancer Research, Section of Immunology, McElwain Laboratories, 15 Cotswold Road, Belmont, Sutton, Surrey SM2 5NG, UK
Received 26 April 1995; revised 9 August 1995; accepted 10 August 1995

were to (a) monitor the toxicity of ICR62 in the dose range of 2.5–100 mg, (b) investigate the localisation of MAb ICR62 to tumour cells in metastatic lesions, (c) determine the dose of ICR62 necessary to achieve circulating ICR62 in the blood and (d) assay for human anti-rat antibody (HARA) responses. This communication describes the results of the first phase I clinical study using a rat MAb to the EGFR.

### Materials and methods

### *Preparation of MAb ICR62 for clinical use*

All antibody for clinical use was prepared according to the guidelines prepared by the Cancer Research Campaign (CRC)-Medical Research Council (MRC) Joint Committee. Hybridoma cells were grown in Dulbecco's modified Eagle medium (DMEM) containing 3% or 5% fetal calf serum of North American origin and antibiotics as described previously (Modjtahedi *et al.*, 1993*a*), in either a Verax Type 1 Bioreactor or as bulk cultures in roller bottles. Supernatants were harvested under aseptic conditions and then precipitated with ammonium sulphate at 45% saturation. Using autoclaved reagents, column packings and containers, the precipitates were dissolved in water and dialysed against 0.0175 M phosphate buffer pH 6.6. After centrifugation in a Beckman 45Ti rotor at 30 000 *g* to remove insoluble material, the dialysate was fractionated by passage through a column of Whatman DE52 cellulose equilibrated with and eluted with 0.0175 M phosphate buffer pH 6.6. The flow through fractions containing the purified (>95% MAb) ICR62 were bulked and dialysed against five changes of sterile phosphate-buffered saline (PBS). After filter sterilisation the preparation was aliquoted, frozen and stored at −20°C until use.

### *Patient details*

Patients were considered eligible for inclusion in this trial who had (i) inoperable histologically or cytologically confirmed diagnosis of squamous cell carcinoma of the lung or head and neck, (ii) immunohistochemically proven tumour expression of the EGFR (iii) an ECOG performance status of 0–2, (iv) no known history of allergy or atopy, (v) no immunological therapy within the previous 4 weeks, (vi) no significant abnormalities of renal, hepatic or bone marrow function (haemoglobin > 10 g $dl^{-1}$, white count > $3 \times 10^9$ $l^{-1}$, platelet > 120, creatinine < 130, liver enzymes and biliru-

**Table I** Patient data

| *Patient no.* | *Sex* | *Site* | *Age* | *PS weight (kg)* | *Previous treatment* | *ICR62 dose* | *CTC toxicity (grade)* | *HARA response* |
|---|---|---|---|---|---|---|---|---|
| 1 | M | HN | 58 | 1<br>46 | S, R, C | 2.5 mg | – | – |
| 2 | F | HN | 74 | 2<br>66 | S, R | 2.5 mg | Fever (I) | – |
| 3 | F | HN | 51 | 1<br>39 | R, C | 2.5 mg | Fever (I)<br>BP (II) | – |
| 4 | M | HN | 49 | 1<br>60 | S, I, C | 10 mg | ALT (I)<br>Fever (I)<br>BP (I)<br>Rigor (I) | – |
| 5 | M | HN | 57 | 1<br>58 | S<br>R<br>C | 10 mg | Rigor (I)<br>Fever (II) | – |
| 6 | M | HN | 46 | 2<br>43 | S, R, C, | 10 mg | N&V (II)<br>BP (I) | – |
| 7 | F | L | 66 | 1<br>55 | – | 20 mg | Rigor (II)<br>Fever (II)<br>Chest pain (I) | – |
| 8 | M | L | 61 | 1<br>71 | 1, S, R, C | 20 mg | Chest pain (I)<br>Fever (I) | – |
| 9 | M | L | 56 | 1<br>61 | R, C, R | 20 mg | Creatinine (I)<br>Fever<br>ALT (I) | +<br>ANTI (ID |
| 10 | F | HN | 75 | 2<br>50 | R, C, | 40 mg | Rigor (I)<br>Fever (I) | – |
| 11 | M | HN | 45 | 1<br>65 | S, R, I, C | 40 mg | ALT (I)<br>Fever (I) | +<br>Anti-ID |
| 12 | F | L | 68 | 1<br>43 | S | 40 mg | Fever (I)<br>Rigor (I)<br>BP (I) | – |
| 13 | M | L | 70 | 1<br>84 | R, I | 100 mg | BP (I)<br>Fever (I)<br>Rigor (I) | – |
| 14 | M | HN | 79 | 1<br>46 | R, I, C | 100 mg | Rigor (II)<br>Fever (I)<br>BP (I) | – |
| 15 | M | L | 65 | 1<br>55 | C, R | 100 mg | Rigor (I)<br>Fever (I) | – |
| 16 | M | HN | 37 | 1<br>79 | S, R, C | 100 mg | Fever (I)<br>Rigor (I) | + |
| 17 | M | L | 67 | 1<br>63 | – | 100 mg | Rigor (I)<br>Nausea (I)<br>Fever (II) | – |
| 18 | M | HN | 44 | 1<br>75 | S, R, C | 100 mg | Rigor (I)<br>Fever (I) | – |
| 19 | F | L | 54 | 0<br>61 | (also breast 1°) | 100 mg | Rigor (II)<br>N&V (II) | + |
| 20 | F | L | 71 | 1<br>96 | | 100 mg | – | – |

g; HN, head and nack; S, surgery; R, radiotherapy; C, chemotherapy; I, immunotherapy; PS, performance status; HARA, human anti-rat antibody; ID, idiotypic.

bin< ×2 normal). The trial had approval from the CRC PhaseI/II Clinical Trials Committee and the Royal Marsden Hospital Ethical Committee and all participants had given written informed consent. Patients were skin tested (10 μg ICR62 intradermally) 1 h before antibody was administered and no patient was found to give an adverse reaction.

Antibody in PBS pH 7.4 was given intravenously as a single bolus injection over a period of 30–60 min. Groups of three patients were treated with 2.5, 10, 20, or 40 mg of ICR62 and a further eight patients were given 100 mg (Table I). All patients were evaluated for toxicity using standard WHO criteria. Blood samples were taken before and at intervals after dosing with ICR62 so that levels of MAb ICR62 in circulation could be measured and sera tested for the presence of HARA. In six patients given antibody doses of 40 mg or 100 mg, biopsies were taken from accessible metastatic lesions 24 h following dosing and examined for the localisation of ICR62.

**Figure 1** Serum levels of MAb ICR62 following dosing with (**a**) 20 mg (patient 8) (**b**) 40 mg (patient 10) (**c**) 100 mg (patient 13) of antibody. Free antibody was determined by inhibition of binding of [$^{125}$I] EGF to EJ cells by doubling dilutions of patients' sera or an ICR62 standard (starting concentration 50 μg ml$^{-1}$).

*Determination of serum levels of ICR62*

The amount of free MAb ICR62 present in serum was determined by its ability to inhibit the binding of [$^{125}$I] EGF to the human bladder carcinoma cell line (EJ) as described previously (Modjtahedi *et al.*, 1993*a*). Doubling dilutions of serum (50 $\mu$l) were mixed with an equal volume of [$^{125}$I] EGF ($4 \times 10^4$ c.p.m.). Standards containing known concentrations of ICR62 were set up in the same way. Aliquots of 90 $\mu$l of each mixture were then transferred to monolayers of EJ cells grown to confluency in 96-well plates. After incubation for 1 h on ice the cells were washed three times, then lysed in 1 M sodium hydroxide containing 1% sarkosyl and the bound radioactivity was determined in a Hydragamma spectrometer (Oakfield Instruments, Oxford).

*Immunohistochemistry*

All immunohistochemical studies were performed by the indirect method using sheep antibodies to rat $F(ab')_2$ conjugated to horseradish peroxidase (Amersham International).

To determine expression of EGFR, tumour biopsies were snap frozen in liquid nitrogen then mounted in OCT medium and sections of 5 $\mu$M thickness cut. One section was stained with haematoxylin and eosin (H&E) for histology. A second sample was fixed in acetone at 4°C for 10 min, then after washing briefly in PBS the section was incubated with MAb ICR62 (100 $\mu$g ml$^{-1}$) for 1 h. Following washing in PBS for 5 min, the sections were incubated with a 1:100 dilution of secondary antibody [sheep anti-rat $F(ab')_2$ conjugated to horse radish peroxidase, Amersham] for 45 min. Peroxidase staining was demonstrated by incubating the sections for 10 min in a solution containing 0.05% diaminobenzidine (Sigma), 0.1% hydrogen peroxide (Merck) and 0.07% imidazole (Merck). After washing in running tap water for 5 min, the sections were counterstained in Mayer's haematoxylin (HD Suppliers) for 30 s. Finally the sections were dehydrated, cleared and mounted.

To assay for the localisation of MAb ICR62 to tumours in patients, biopsies were taken from an accessible metastatic site 24 h after antibody dosing. Serial sections were cut and stained with secondary antibody alone or ICR62 followed by the secondary antibody. This technique enabled us to determine the proportion of EGFR-expressing cells that had bound administered ICR62 (Modjtahedi *et al.*, 1994).

*Assay for human anti-rat (HARA) response*

We have investigated the immunogenicity of MAb ICR62 by determining if human anti-rat antibodies were present in the sera of patients given different doses of ICR62. Also, we have investigated if the human anti-rat response included anti-idiotypic antibodies by determining if the antibodies in the patient's sera bound to scFv fragments of ICR62. Polyvinyl chloride 96-well plates (Dynatech Labs, VA, USA), were coated with rat antibody by incubation overnight at 4°C with 50 $\mu$l per well of a stock solution (10 $\mu$g 5 ml$^{-1}$ of PBS) of ICR62, ICR62 Fab (Modjtahedi *et al.*, 1995), or ICR62 scFv (C Dean *et al.*, manuscript in preparation). The plates were washed three times with PBS containing 0.5% bovine serum albumin (BSA) and then incubated for 2 h with 200 $\mu$l per well of PBS–0.5% BSA to block the remaining sites. After a further three washes with PBS containing 0.5% BSA, doubling dilutions of the patient's sera in PBS–0.5% BSA were added in duplicate to the wells and the plates were incubated for 1 h at ambient temperature. After washing the plates three times with PBS–0.5% BSA, human antibodies bound to ICR62 or its fragments were detected by the addition of $^{125}$I-labelled rabbit anti-human $F(ab')_2$. After incubation for 1 h at ambient temperature, the plates were washed three times with PBS – 0.5% BSA and then cut into individual wells and the bound radioactivity determined.

### Results

*Patient details and clinical observations*

Twenty patients, 11 with head and neck cancers and nine with lung cancers were recruited. Patient details are summarised in Table I. Following intravenous injections of the antibody, 18 patients exhibited mild (WHO grade I–II) rigors and fever or hypotension (Table I). Symptoms were controlled by hydrocortisone (100 mg) and Piriton (10 mg). In no case were any severe toxicities (Grade III–IV) observed. None of the patients reported any untoward effects of their treatment during the follow-up period.

*Serum levels of ICR62*

No free antibody was detected in the sera, sampled at 4 h or 24 h post dosing, of patients given 20 mg or less of ICR62 (Figure 1a). However, MAb ICR62 could be detected in the sera of patients following doses of 40 mg or 100 mg (Figure 1b and 1c). In addition, the level of ICR62 remaining in circulation was found to be highest in the patients receiving 100 mg of ICR62 (Figure 1). In one such patient (number 13), MAb ICR62 was readily detectable in the serum at a

**Figure 2** Localisation of MAb ICR62 to metastatic lesions. (**a**) Frozen section of a squamous cell carcinoma from patient 12 taken 24 h following dosing with 40 mg of ICR62 and stained with peroxidase-conjugated secondary antibody only. The ICR62 antibody has localised to a peripheral rim of the epithelium about 1–3 cells deep. (**b**) Consecutive section from the same block stained with ICR62 followed by secondary antibody showing that all cells of the epithelial component express EGFR. Magnification ×26.

**Figure 3** Localisation of MAb ICR62 to metastatic lesions. Frozen section of a squamous carcinoma from a metastatic lesion from patient 18 taken 24 h following dosing with 100 mg of ICR62 and stained with peroxidase-conjugated secondary antibody only (**a**), (**b**). Consecutive section from the same block stained with ICR62 and the peroxidase-conjugated secondary antibody showing that all epithelial cells express the EGFR (**c**), (**d**). Magnification (**a**) and (**c**) (×26), (**b**) and (**d**) different fields in (**a**) and (**c**) at ×130.

concentration of about 8 $\mu$g ml$^{-1}$, 3 days following administration (Figure 1c). No free MAb could be detected in the serum 7 days post ICR62 treatment in any patient.

*Injected ICR62 binds to the EGFR on tumour cell membranes*

Having shown that MAb ICR62 was in circulation at 24 h in patients given doses of 40 mg, we subsequently investigated whether in such patients MAb ICR62 was localised to the tumour. Biopsies of metastatic lesions were taken from six patients, 24 h after ICR62 dosing. Two of the biopsies (patients 17 and 19) were found to consist largely of necrotic material and were discarded, but four (patients 10, 12, 18 and 20) yielded well-defined samples containing viable tumour. The sections illustrated in Figure 2a show that 24 h after treatment of patient 12 with 40 mg of ICR62, the antibody had localised to the membranes of tumour cells around the periphery of the epithelial cell islands in the metastatic site. When a sequential section was stained with ICR62 before the addition of the sheep anti-rat reagent, it was found that all cells, including those in the interior of the epithelial cell islands expressed the EGFR (Figure 2b).

However, when frozen sections of biopsies obtained from metastases of patients treated with 100 mg of ICR62 were examined, it was clear that the MAb had penetrated further into the epithelial component of the metastatic lesions with a greater proportion of EGFR-positive cells having bound administered ICR62 (e.g. Figures 3a and 3c or 3b and 3d).

*Development of human antibodies to ICR62*

Of the 20 patients treated with ICR62, HARA were detected in the sera of four patients (numbers 9, 11, 16 and 19; see Table I). Of these, only two patients given 20 mg (no. 9) or 40 mg (no. 11) produced anti-idiotypic antibodies that bound to scFv ICR62 (Figure 4). Sera from two out of eight patients treated with 100 mg of ICR62 (patients 16 and 19) contained antibodies directed against determinants on the constant region since they bound to the Fab fragment and intact antibody but not to the ICR62 scFv (Table I, Figure 5). In one patient (no. 12), the results obtained with the serum taken before treatment indicated that antibodies that bound to rat IgG were present in the sera before ICR62 was administered. It is unknown whether binding was specific or due to the presence of cross-reactive autoantibodies.

### Discussion

Overexpression of the EGFR accompanied by production of the EGF family of ligands has been found to occur in a wide range of human malignancies and this phenomenon has been correlated with a poorer prognosis in these patients (for review see Gullick, 1991; Modjtahedi and Dean, 1994). During the past 14 years a number of mouse monoclonal antibodies have been raised against epitopes on the external domain of the human EGFR and these have been used not only to investigate growth factor–receptor interaction and the mechanism(s) of activation of the EGF receptor system

**Figure 4** Development of human anti-rat antibodies in the serum of patient number 9 following injection of 20 mg ICR62 shown by binding to intact ICR62 (**a**), Fab ICR62 (**b**) and scFv ICR62 (**c**). This indicates an anti-idiotypic specificity, ■ Prebleed; □, day14; ◆, day 28.

**Figure 5** Development of human anti-rat antibodies in the serum of patient number 11 following injection of 40 mg of ICR62 shown by binding to intact ICR62 (**a**), Fab ICR62 (**b**) but not scFv ICR62 (**c**). This indicates non-idiotypic (constant region) specificity. ■, Prebleed; □, day 8; ◆, day 15.

but also for diagnostic and therapeutic applications in cancer (for review see Modjtahedi and Dean, 1994). Several of the mouse antibodies have undergone clinical evaluation in phase I and phase II studies in patients with head and neck, lung or brain cancers including MAb EGFR1 (Soo *et al.*, 1987; Kalofonos *et al.*, 1989), MAbs 225 and 528 (Divigi *et al.*, 1991; Baselga *et al.*, 1993), MAb 425 (EMD 55900 E Merck, Brady *et al.*, 1991; Magdelenat *et al.*, 1991; Stasiecki *et al.*, 1993; Blizer *et al.*, 1993; Dadparvar *et al.*, 1994) and MAb RG83852 (Perez-Soler *et al.*, 1994). The aim of these studies, in common with the one presented here, was to determine whether treatment of cancer patients with anti-EGFR MAbs produced life-threatening toxicities by their binding to EGFR expressed by normal tissues, including liver and skin. The results of these studies have shown that mouse antibodies to the EGFR can be given safely to patients without untoward toxicity. For example, Divigi *et al.* (1991) have treated patients with advanced squamous cell carcinoma of the lung with single doses of up to 300 mg of MAb 225, including 4 mg of $^{111}$In-labelled 225, without significant toxicity. Furthermore, with doses of 40 mg or more they were able to image presumed sites of metastasis greater than or equal to 1 cm in diameter.

Immunohistological examination of the biopsies from metastatic sites, taken 24 h following ICR62 dosing, demonstrated the localisation of MAb ICR62 to the tumour cells. There appeared to be a dose–response effect in that the depth of tumour penetration at a dose of 100 mg exceeded that at 40 mg. The good tumour localisation of ICR62 in the biopsies from patients treated with 100 mg of antibody was encouraging. Indeed, the results of another study using the mouse antibody RG83852 showed that localisation could only be detected at doses of 400 mg $m^{-2}$ or greater (Perez-Soler *et al.*, 1994). We selected MAb ICR62 for a phase I clinical study since it was the most effective of a number of rat MAbs we had generated against the human EGFR at inducing the regression of xenografts of head and neck, vulva and breast carcinomas grown in athymic mice (Modjtahedi *et al.*, 1993*b*, 1994; Dean *et al.*, 1994).

In the present study, the results of the immunohistological investigation showed that the cells adjacent to the vasculature were strongly stained. This tumour cell population is likely to have the highest proliferation index and any antiproliferative/pro-differentiation effect here may be therapeutically significant. This study has shown that MAb ICR62 can be given safely to cancer patients at doses up to 100 mg producing only mild toxicity. This trial was not designed to test the anti-tumour activity of this agent.

The maintainance of receptor blockade may require repeated treatment with antibody. Certainly, our experience using xenograft models (Modjtahedi *et al.*, 1993b, 1994) points to the need to maintain sufficiently high blood levels for long enough to (a) block EGFR function, (b) recruit to the tumour and activate host effector cells and (c) induce terminal differentiation. The limited data on the serum half-life of ICR62 obtained in this investigation indicate that free ICR62 was present 2–3 days post treatment and therefore suggest that twice weekly doses of 100 mg may be sufficient to maintain a high enough level of this antibody for therapeutic activity. In the present investigation we were able to biopsy metastatic sites at only a single early time point so we have no information concerning the stability of the antibody at the tumour cell surface or of the effects of treatment on the recruitment and activation of host immune effector cells. Such a study will form part of the next clinical trial, in which we propose to investigate the potential therapeutic effect of multiple treatments with ICR62.

Just 4 of the 20 patients developed HARA following a single dose of MAb ICR62. Of these, only two were directed against the idiotype of ICR62. These results suggest that

MAb ICR62 is not as immunogenic as the mouse antibodies to EGFR previously used in clinical studies. For example, Divigi *et al.* (1991) found all 19 lung cancer patients treated with a single dose of 1–300 mg of MAb 225 developed human anti-mouse antibodies. Stasieki *et al.* (1993) have also found that a single infusion or multiple infusions at monthly intervals of MAb EMD 55900 in glioma patients elicited human anti-mouse antibodies. On the other hand, these authors reported that following multiple infusions of glioma patients with MAb EMD 55900 at shorter intervals (three times per week, during 4 weeks or longer), human anti-mouse antibodies were not detectable in the sera of these patients (Stasiecki *et al.*, 1993). In another encouraging clinical trial reported recently, Riethmuller *et al.* (1994) have shown that adjuvant treatment of 189 patients (Dukes' C colorectal cancer) with 500 mg of mouse antibody 17-1A followed by four 100 mg infusions at monthly intervals induced HAMA responses in 80% of treated patients. However despite this, the treatment schedule reduced the overall 5 year death rate by 30% and decreased the recurrence rate by 27%. If HARA developed following repeated doses of ICR62 and compromised potential therapeutic effects due to rapid clearance, this problem may be reduced by use of either chimaeric or humanised versions of the antibodies. Alternatively, we have the benefit of a variety of EGFR MAbs, which recognise different epitopes on the EGFR and are of different isotypes, that could be used for second and subsequent treatments (Modjtahedi and Dean, 1994).

In summary, the results of this phase I clinical study with the limited amounts of MAb ICR62 available have indicated that MAb ICR62, which acts as an EGF, TGF-$\alpha$ and HB-EGF antagonist, (a) can be administered safely to patients with squamous cell carcinoma; (b) localises efficiently to metastatic sites; and (c) may therefore have potential for the treatment of the significant number of cancer patients whose tumours overexpress the EGF receptor. A further clinical study is planned using higher doses of MAb ICR62 given either singly or as multiple doses.

**Acknowledgements**
This work was supported by grants from the Medical Research Council and Cancer Research Campaign, London.

### References

AARONSON SA. (1991). Growth factors and cancer. *Science*, **254**, 1146–1153.

BASELGA J AND MENDELSOHN J. (1994). The epidermal growth factor receptor as a target for therapy in breast carcinoma. *Breast Cancer Res. Treat.*, **29**, 127–138.

BASELGA J, SCOTT A, PFISTER D, KRIS M, DIVIGI C, ZHANG Z, LARSONS, OETTGEN H AND MENDELSOHN J. (1993). Comparative pharmacology in Phase I and imaging trials utilizing anti-epidermal growth factor receptor (EGFR) monoclonal antibodies (MAbs) labeled with $^{131}$I or $^{111}$Indium. *Proc. Ann. Meet. Am. Soc. Clin. Oncol.*, **12**, A368.

BILZER T, STASSIECKI P, VEGA F, KEMSBEAD JT, WESTPHAL M, SSHNUM E AND WECHSLER W. (1993). Immunotherapy of malignant gliomas with the anti-EGFR monoclonal antibody. *Proc. Ann. Meet. Am. Assoc. Cancer Res.*, **34**, A2877.

BRADY LW, MIYAMOTO C, WOO DV, RACKOVER M, EMRICH J, BENDER H, DADPARVAR S, STEPLEWSKI Z, KAPROWSKI H, BLACK P, LAZZARO B, NAIR S, MCCOROMACK T, NIEVES J, MARABITO M AND ESHLWMAN J. (1991). Malignant astrocytomas treated with iodine-125 labelled monoclonal antibody 425 against epidermal growth factor receptor: a phase II trial. *Int. J. Radiat. Oncol. Biol. Phys.*, **22**, 225–230.

CARPENTER G. (1987). Receptor for epidermal growth factor and other polypeptide mitogens. *Annu. Rev. Biochem.*, **56**, 881–914.

COHEN S. (1962). Isolation of a mouse submaxillary gland protein accelerating incisor eruption and eyelid opening in the new-born animal. *J. Biol. Chem.*, **237**, 1555–1562.

COWLEY G, SMITH JA AND GUSTERSON B. (1984). The amount of EGF receptor is elevated on squamous cell carcinomas. *Cancer Cells*, **1**, 5–10.

CROSS M AND DEXTER TM. (1991). Growth factors in development, transformation, and tumorigenesis. *Cell*, **64**, 271–280.

DADPARVAR S, KRISHNA L, MIYAMOTO C, BRADY LW, BROWN SJ, BENDER H, SLIZOFSKI WJ, CHEVRES A AND WOO DV. (1994). Indium-111-labeled anti-EGFr-425 scintigraphy in the detection of malignant gliomas. *Cancer Suppl.*, **73**, 884–889.

DAS M, MIKYAWA T, FOX CF, PRUSS RM, AHARONOV A AND HERSHAMAN HR. (1977). Specific radiolabelling of cell surface receptor for EGF. *Proc. Natl Acad. Sci. USA*, **74**, 2790–2794.

DEAN CJ, MODJTAHEDI H, ECCLES SA, BOX G AND STYLES JM. (1994). Immunotherapy with antibodies to the EGF receptor. *Int. J. Cancer*, **8**, 103–107.

DERYNCK R. (1992). The physiology of transforming growth factor $\alpha$. *Adv. Cancer Res.*, **58**, 27–52.

DIVIGI CR, WEST S, KRIS M, REAL FX, YEH DJ, GRALLA R, MERCHANT B, SCHWEIGHT S, UNGER M, LARSON SM AND MENDELSOHN J. (1991). Phase I and imaging trial of Indium III-labelled anti-EGF receptor antibody 225 in patients with squamous cell lung carcinomas. *J. Natl Cancer Inst.*, **83**, 97–104.

GOUSTIN AS, LEOF EB, SHIPLEY GD AND MOSES H. (1986). Growth factors and cancer. *Cancer Res.*, **46**, 1015–1029.

GULLICK WJ. (1991). Prevalence of aberrant expression of the epidermal growth factor receptor in human cancers. *Br. Med. Bull.*, **47**, 87–98.

HARRIS AL. (1994). What is the biological, prognostic, and therapeutic role of the EGF receptor in human breast cancer? *Breast Cancer Res. Treat.*, **29**, 1–2.

HIGASHIYAMA S, ABRAHAM JA AND MILLER J. (1991). A heparin-binding growth factor secreted by macrophage-like cells that is related to EGF. *Science*, **257**, 936–939.

KALOFONOS HP, PAWLIKOWSKA TR, HEMINGWAY A, COURTNAY-LUCK N, DHOKIA B, SNOOK D, SIVALAPENKO GB, HOOKER JR, MCKENZIE CG, LAVENDER PJ, THOMAS DCT AND EPENETOS AA. (1989). Antibody guided diagnosis and therapy of brain gliomas using radiolabeled monoclonal antibodies against epidermal growth factor receptor and placental alkaline phosphatase. *J. Nucl. Med.*, **30**, 1636–1645.

MAGDELENAT H, DELATTRE JY, MADY E, FAOLLOT T, VEGA F AND POISSON M. (1991). A phase I study of the anti-EGFR monoclonal antibody 425 in patients with malignant gliomas. *J. Tumor Marker Oncol.*, **6(4)**, 60.

MENDELSOHN J AND LIPPMAN, ME. (1993). Principles of molecular cell biology of cancer: Growth factors. In *Cancer: Principles & Practice of Oncology*, DeVita Jr VT, Hellman S and Rosenberg SA (eds) pp 114–133. JB Lippincott: Philadelphia.

MODJTAHEDI H AND DEAN CJ. (1994). The receptor for EGF and its ligands: Expression prognostic value and target for therapy in cancer (review). *Int. J. Oncol.*, **4**, 277–296.

MODJTAHEDI H AND DEAN C. (1995). The binding of HG-EGF to tumour cells is blocked by MAbs which act as EGF and TGF$\alpha$ antagonists. *Biochem. Biophys. Res. Commun.*, **207**, 389–397.

MODJTAHEDI H, STYLES JM AND DEAN CJ. (1993*a*). The human EGF receptor as a target for cancer therapy: six new rat MAbs against the receptor on the breast carcinoma MDA-MB 468. *Br. J. Cancer*, **67**, 247–253.

MODJTAHEDI H, ECCLES SA, BOX G, STYLES JM AND DEAN CJ. (1993*b*). Immunotherapy of human tumour xenografts overexpressing the EGF receptor with rat antibodies that block growth factor-receptor interaction. *Br. J. Cancer*, **67**, 254–261.

MODJTAHEDI H, ECCLES SA, SANDLE J, BOX G, TITLEY J AND DEAN CJ. (1994). Differentiation or immune destruction: two pathways for therapy of squamous cell carcinomas with antibodies to the epidermal growth factor receptor. *Cancer Res.*, **54**, 1695–1701.

MODJTAHEDI H, JACKSON E AND DEAN C. (1995). Monovalent antibodies to the EGF receptor. Effects on proliferation and differentiation of tumours overexpressing the EGF receptor. *Tumour Targeting*, **1**, 99–106.

NEAL DE, SHARPLES L AND SMITH K. (1990). The epidermal growth factor and the prognosis of bladder cancer. *Cancer*, **65**, 1619–1625.

NICOLSON GL. (1993). Cancer progression and growth: relationship of paracrine and autocrine growth mechanisms to organ preference of metastasis. *Exp. Cell Res.*, **204,** 171-180.

OZANNE BW, RICHARDS CS, HENDELER F, BURNS D AND GUSTERSON B. (1986). Overexpression of the EGF receptor is a hallmark of squamous cell carcinomas. *J. Pathol.*, **149,** 9-14. 1.

PEREZ-SOLER R, DONATO NJ, SHIN DM, ROSENBLUM MG, ZHANG HU, TORNOS G, BREWER H, CHANG JC, LEE JS, HONG WK AND MURRAY JL. (1994). Tumour epidermal growth factor receptor studies in patients with non-small-cell lung cancer or head and neck cancer treated with monoclonal antibody RG 83852. *J. Clin. Oncol.*, **12,** 730-739.

PUSZTAI L, LEWIS CE, LORENZEN J AND MCGEE OD. (1993). Growth factors: regulation of normal and neoplastic growth. *J. Pathol.*, **169,** 191-201.

RIETHMULLER G, SCHNEIDER-GADICKE E, SCHLIMOK G, SCHMIEGEL W, RAAB R, HÖFFKEN K, GRUBER R, PICHLMAIER H, HIRCHE H, PICHLMAYR R, BUGGISCH P, WITTE J AND the German Cancer Aid 17-1A Study Group (1994). Randomised trial of monoclonal antibody for adjuvant therapy of resected Dukes's colorectal carcinoma. *Lancet*, **343,** 1177-1183.

SHING Y, CHRISTOFORI G AND HANAHAN D. (1993). Betacellulin: a mitogen from pancreatic $\beta$ cell tumors. *Science*, **259,** 1604-1607.

SHOYAB M, PLOWMAN GD AND MCDONALD VL. (1989). Structure and function of human amphiregulin: a member of the epidermal growth factor family. *Science*, **243,** 1074-1076.

SOO KC, WARD M, ROBERTS KR, KEELING F, CARTER RL, MCREADY VR, OTT RJ, POWELL E, OZANNE B, WESTWOOD JH AND GUSTERSON BA. (1987). Radioimmunoscintigraphy of squamous carcinomas of the head and neck. *Head and Neck Surgery*, **9,** 349-352.

SPORN MB AND ROBERTS AB. (1985). Autocrine growth factors and cancers. *Nature*, **313,** 745-747.

STASIECKI P, KEMSHEAD JT, WESTPHAL, M, DELATTRE JY, GROPP P AND SIMANE Z. (1993). The development of HAMA in glioma patients can be suppressed by administration of murine MAb in short time intervals. *Proc. Ann. Meet. Am. Assoc. Cancer Res.*, **34,** A2835.

THOMPSON DM AND GILL GN. (1985). The EGF receptor: Structure regulation and potential role in malignancy. *Cancer Surveys*, **4,** 767-788.

TODARO GJ, DE LARCO JE AND COHEN S. (1976). Transformation by murine and feline sarcoma viruses specifically blocks binding of epidermal growth factor to cells. *Nature*, **264,** 26-31.

VAN DE VIJVERT M, KUMAR R AND MENDELSOHN J. (1991). Ligand-induced activation of A431 cell epidermal growth factor occurs primarily by an autocrine pathway that acts upon receptors on the surface rather than intracellularly. *J. Biol Chem.*, **266,** 7503-7508.

WATERFIELD MD. (1991). The role of growth factors in cancer. In *Introduction to the Cellular & Molecular Biology of Cancer*, second edn Franks LM and Teich NM (eds) pp. 296-329. Oxford University Press: Oxford.


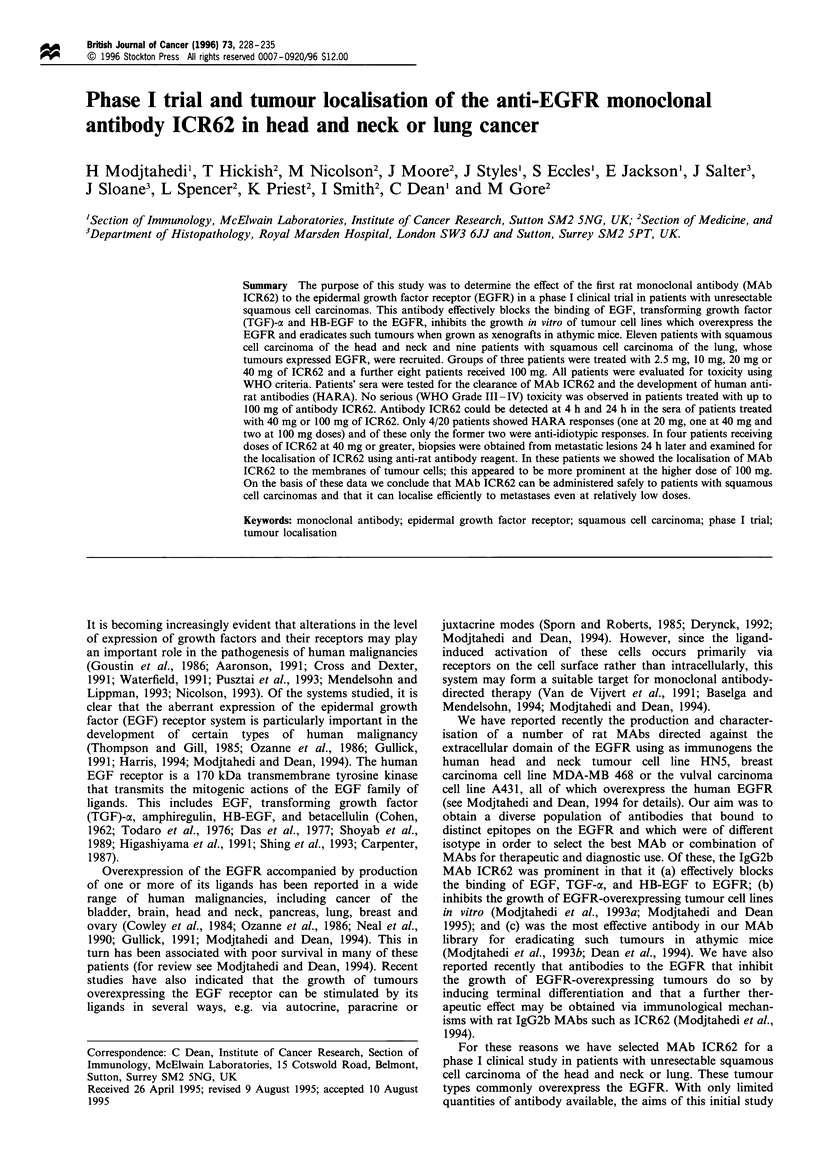

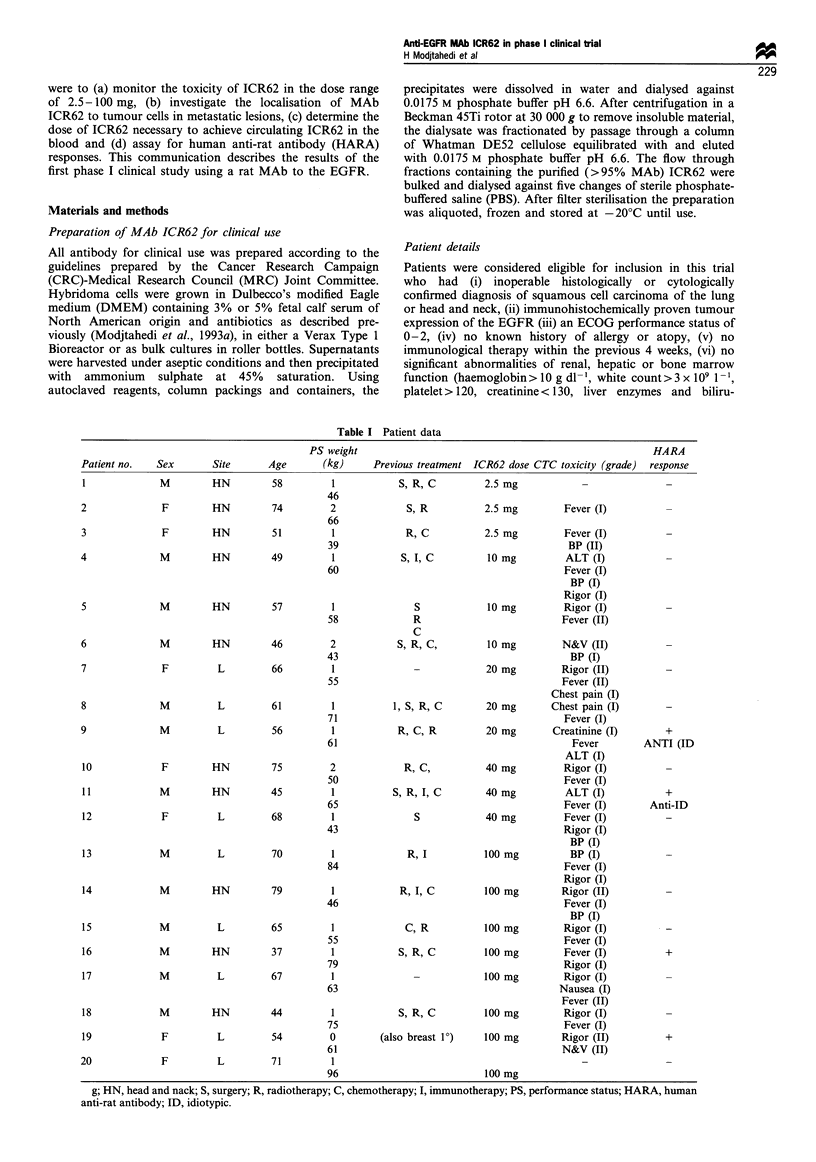

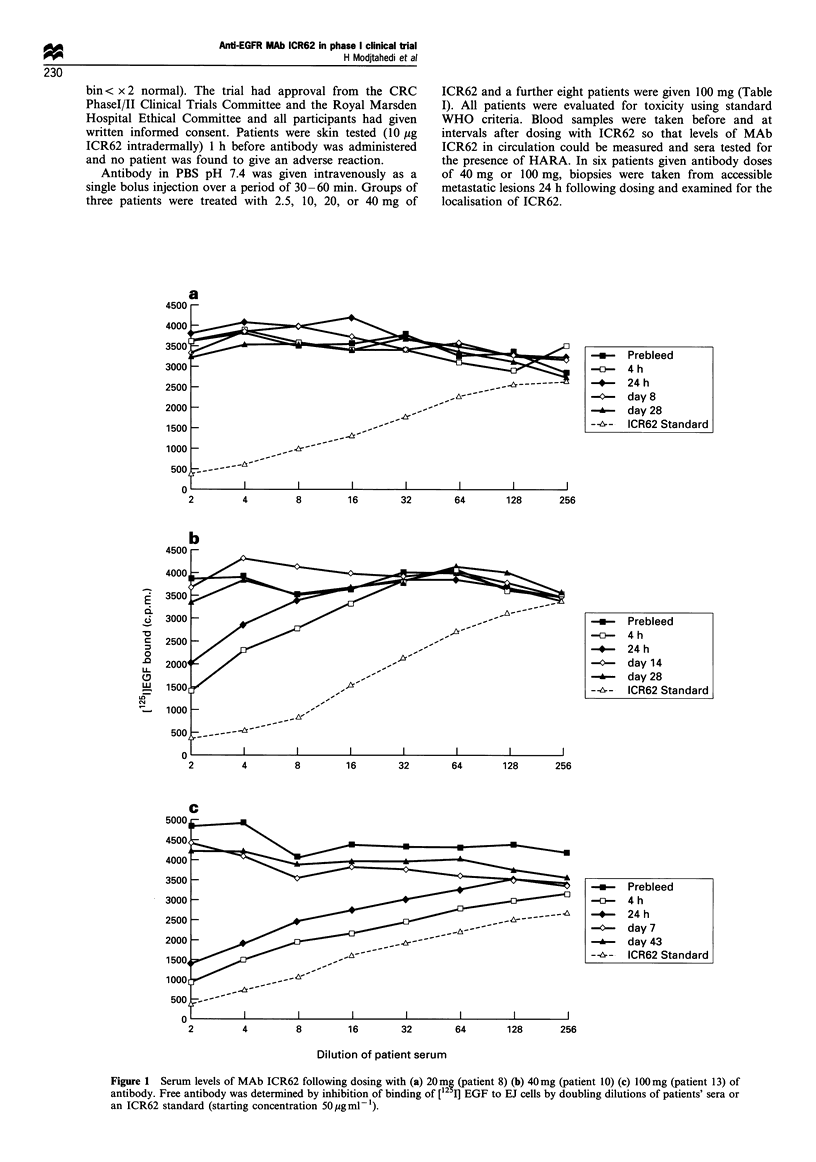

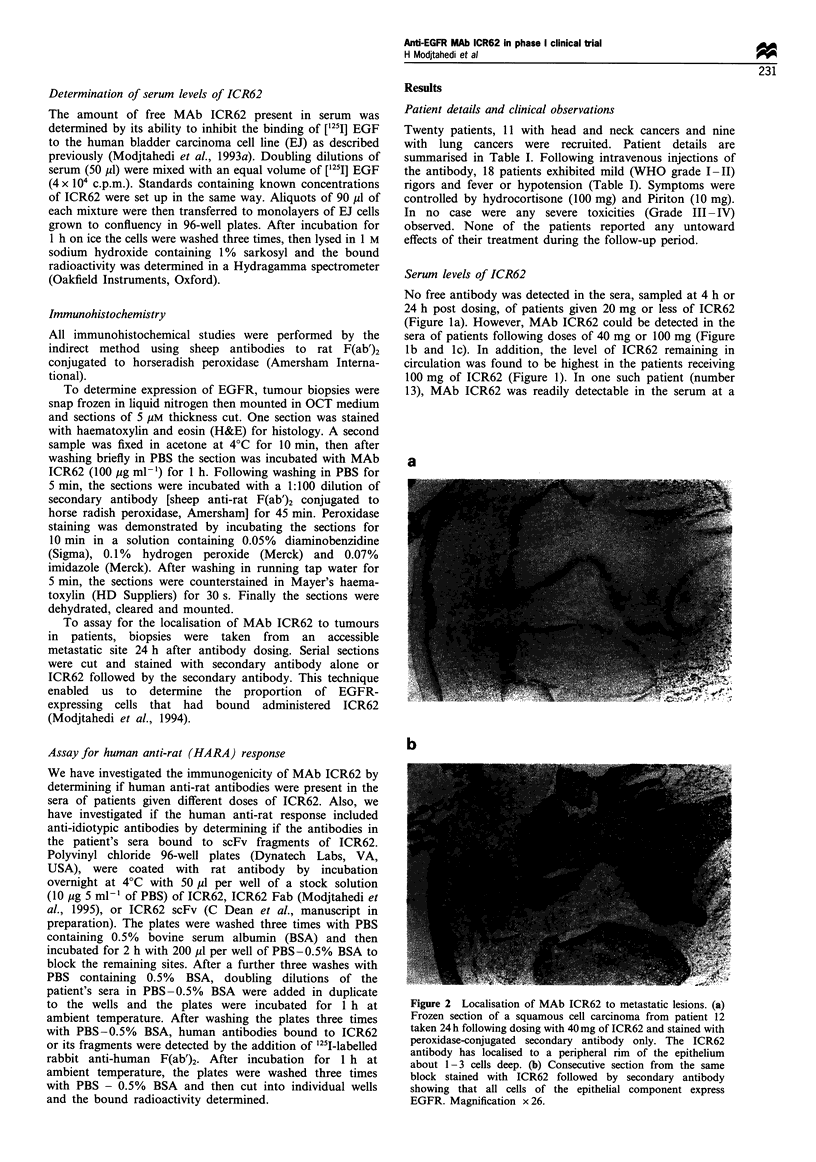

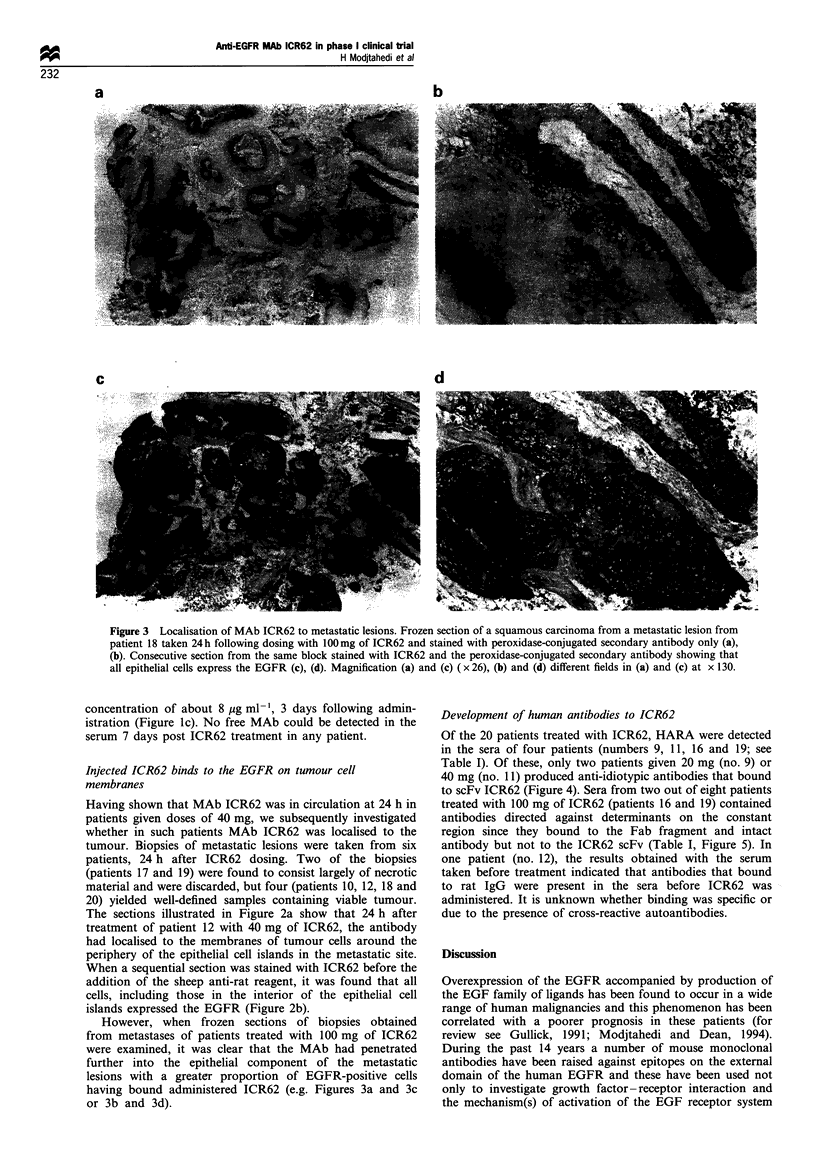

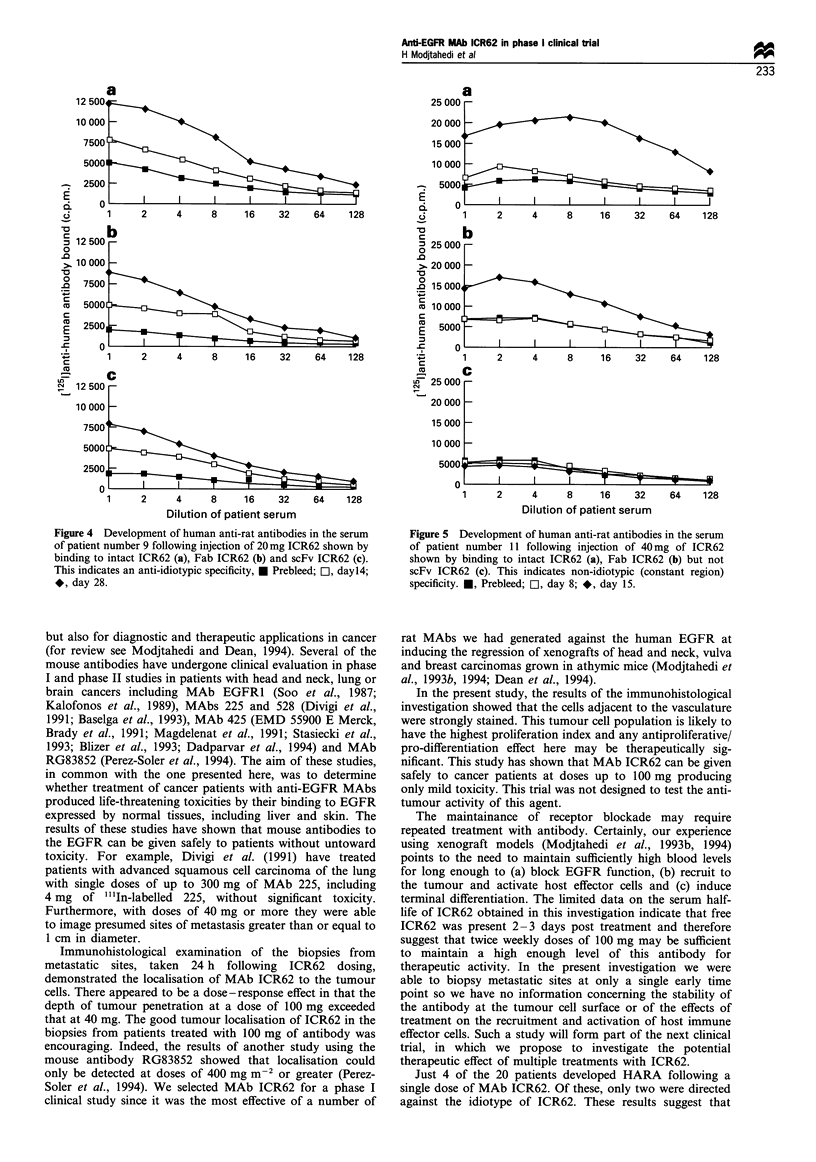

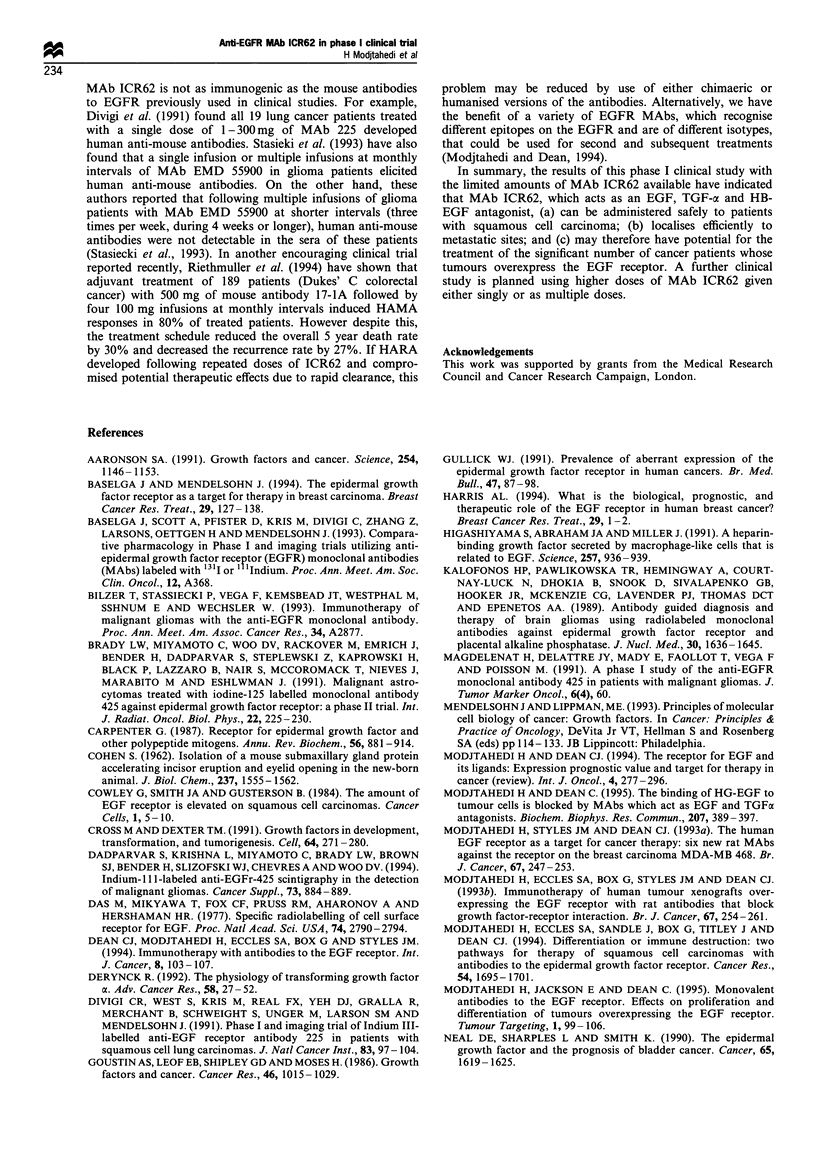

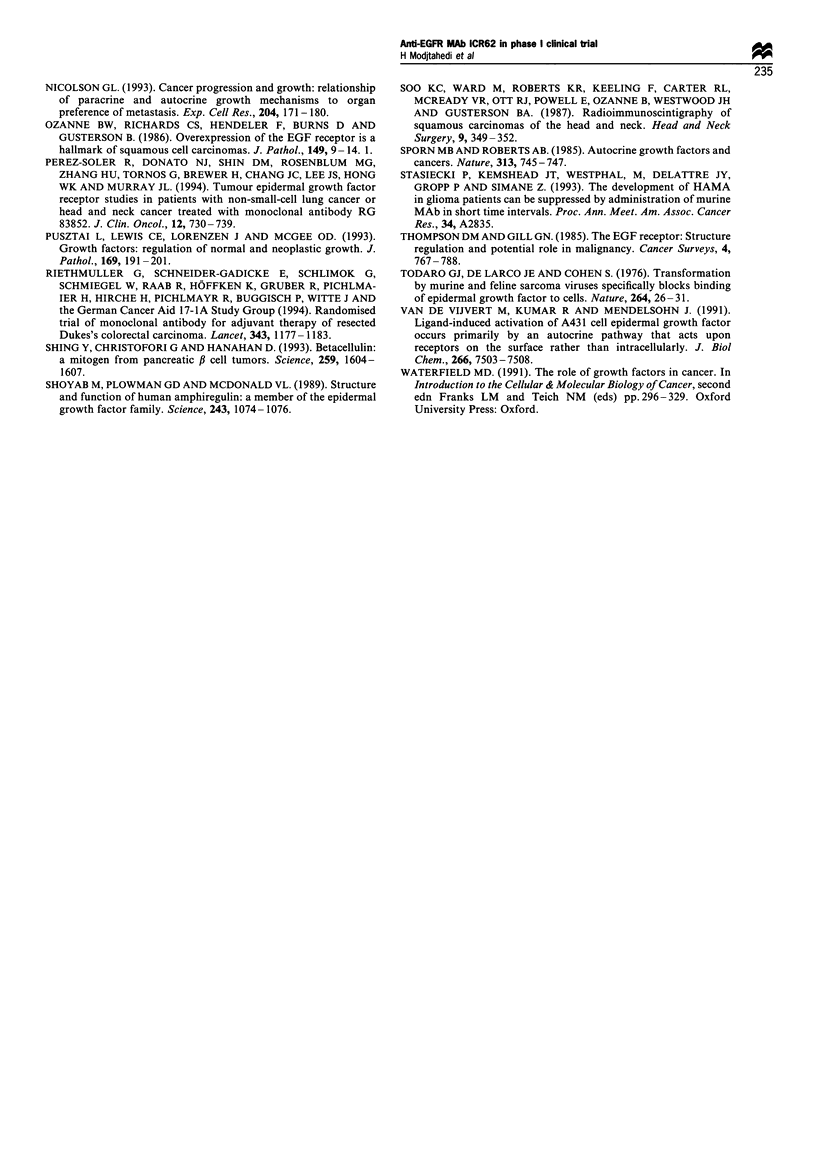


## References

[OCR_00876] Aaronson S. A. (1991). Growth factors and cancer.. Science.

[OCR_00880] Baselga J., Mendelsohn J. (1994). The epidermal growth factor receptor as a target for therapy in breast carcinoma.. Breast Cancer Res Treat.

[OCR_00899] Brady L. W., Miyamoto C., Woo D. V., Rackover M., Emrich J., Bender H., Dadparvar S., Steplewski Z., Koprowski H., Black P. (1992). Malignant astrocytomas treated with iodine-125 labeled monoclonal antibody 425 against epidermal growth factor receptor: a phase II trial.. Int J Radiat Oncol Biol Phys.

[OCR_00910] COHEN S. (1962). Isolation of a mouse submaxillary gland protein accelerating incisor eruption and eyelid opening in the new-born animal.. J Biol Chem.

[OCR_00906] Carpenter G. (1987). Receptors for epidermal growth factor and other polypeptide mitogens.. Annu Rev Biochem.

[OCR_00922] Cross M., Dexter T. M. (1991). Growth factors in development, transformation, and tumorigenesis.. Cell.

[OCR_00924] Dadparvar S., Krishna L., Miyamoto C., Brady L. W., Brown S. J., Bender H., Slizofski W. J., Eshleman J., Chevres A., Woo D. V. (1994). Indium-111-labeled anti-EGFr-425 scintigraphy in the detection of malignant gliomas.. Cancer.

[OCR_00930] Das M., Miyakawa T., Fox C. F., Pruss R. M., Aharonov A., Herschman H. R. (1977). Specific radiolabeling of a cell surface receptor for epidermal growth factor.. Proc Natl Acad Sci U S A.

[OCR_00937] Dean C., Modjtahedi H., Eccles S., Box G., Styles J. (1994). Immunotherapy with antibodies to the EGF receptor.. Int J Cancer Suppl.

[OCR_00940] Derynck R. (1992). The physiology of transforming growth factor-alpha.. Adv Cancer Res.

[OCR_00947] Divgi C. R., Welt S., Kris M., Real F. X., Yeh S. D., Gralla R., Merchant B., Schweighart S., Unger M., Larson S. M. (1991). Phase I and imaging trial of indium 111-labeled anti-epidermal growth factor receptor monoclonal antibody 225 in patients with squamous cell lung carcinoma.. J Natl Cancer Inst.

[OCR_00950] Goustin A. S., Leof E. B., Shipley G. D., Moses H. L. (1986). Growth factors and cancer.. Cancer Res.

[OCR_00956] Gullick W. J. (1991). Prevalence of aberrant expression of the epidermal growth factor receptor in human cancers.. Br Med Bull.

[OCR_00964] Higashiyama S., Abraham J. A., Miller J., Fiddes J. C., Klagsbrun M. (1991). A heparin-binding growth factor secreted by macrophage-like cells that is related to EGF.. Science.

[OCR_00973] Kalofonos H. P., Pawlikowska T. R., Hemingway A., Courtenay-Luck N., Dhokia B., Snook D., Sivolapenko G. B., Hooker G. R., McKenzie C. G., Lavender P. J. (1989). Antibody guided diagnosis and therapy of brain gliomas using radiolabeled monoclonal antibodies against epidermal growth factor receptor and placental alkaline phosphatase.. J Nucl Med.

[OCR_00997] Modjtahedi H., Dean C. (1995). The binding of HB-EGF to tumour cells is blocked by mAbs which act as EGF and TGF alpha antagonists.. Biochem Biophys Res Commun.

[OCR_01008] Modjtahedi H., Eccles S., Box G., Styles J., Dean C. (1993). Immunotherapy of human tumour xenografts overexpressing the EGF receptor with rat antibodies that block growth factor-receptor interaction.. Br J Cancer.

[OCR_01014] Modjtahedi H., Eccles S., Sandle J., Box G., Titley J., Dean C. (1994). Differentiation or immune destruction: two pathways for therapy of squamous cell carcinomas with antibodies to the epidermal growth factor receptor.. Cancer Res.

[OCR_01002] Modjtahedi H., Styles J. M., Dean C. J. (1993). The human EGF receptor as a target for cancer therapy: six new rat mAbs against the receptor on the breast carcinoma MDA-MB 468.. Br J Cancer.

[OCR_01027] Neal D. E., Sharples L., Smith K., Fennelly J., Hall R. R., Harris A. L. (1990). The epidermal growth factor receptor and the prognosis of bladder cancer.. Cancer.

[OCR_01032] Nicolson G. L. (1993). Cancer progression and growth: relationship of paracrine and autocrine growth mechanisms to organ preference of metastasis.. Exp Cell Res.

[OCR_01037] Ozanne B., Richards C. S., Hendler F., Burns D., Gusterson B. (1986). Over-expression of the EGF receptor is a hallmark of squamous cell carcinomas.. J Pathol.

[OCR_01043] Perez-Soler R., Donato N. J., Shin D. M., Rosenblum M. G., Zhang H. Z., Tornos C., Brewer H., Chan J. C., Lee J. S., Hong W. K. (1994). Tumor epidermal growth factor receptor studies in patients with non-small-cell lung cancer or head and neck cancer treated with monoclonal antibody RG 83852.. J Clin Oncol.

[OCR_01050] Pusztai L., Lewis C. E., Lorenzen J., McGee J. O. (1993). Growth factors: regulation of normal and neoplastic growth.. J Pathol.

[OCR_01055] Riethmüller G., Schneider-Gädicke E., Schlimok G., Schmiegel W., Raab R., Höffken K., Gruber R., Pichlmaier H., Hirche H., Pichlmayr R. (1994). Randomised trial of monoclonal antibody for adjuvant therapy of resected Dukes' C colorectal carcinoma. German Cancer Aid 17-1A Study Group.. Lancet.

[OCR_01061] Shing Y., Christofori G., Hanahan D., Ono Y., Sasada R., Igarashi K., Folkman J. (1993). Betacellulin: a mitogen from pancreatic beta cell tumors.. Science.

[OCR_01066] Shoyab M., Plowman G. D., McDonald V. L., Bradley J. G., Todaro G. J. (1989). Structure and function of human amphiregulin: a member of the epidermal growth factor family.. Science.

[OCR_01077] Soo K. C., Ward M., Roberts K. R., Keeling F., Carter R. L., McCready V. R., Ott R. J., Powell E., Ozanne B., Westwood J. H. (1987). Radioimmunoscintigraphy of squamous carcinomas of the head and neck.. Head Neck Surg.

[OCR_01082] Sporn M. B., Roberts A. B. Autocrine growth factors and cancer.. Nature.

[OCR_01095] Thompson D. M., Gill G. N. (1985). The EGF receptor: structure, regulation and potential role in malignancy.. Cancer Surv.

[OCR_01100] Todaro G. J., De Larco J. E., Cohen S. (1976). Transformation by murine and feline sarcoma viruses specifically blocks binding of epidermal growth factor to cells.. Nature.

[OCR_01103] Van de Vijver M. J., Kumar R., Mendelsohn J. (1991). Ligand-induced activation of A431 cell epidermal growth factor receptors occurs primarily by an autocrine pathway that acts upon receptors on the surface rather than intracellularly.. J Biol Chem.

